# Supplementary material for: Orthosteric STING inhibition elucidates molecular correction of SAVI STING
Source: Nat Commun. 2025 Jul 1;16:5695. doi: 10.1038/s41467-025-60632-5 (PMC12217682; doi:10.1038/s41467-025-60632-5)

Orthosteric STING inhibition elucidates molecular correction of SAVI STING

Tao Xie[1,†], Max Ruzanov[2], David Critton[2], Leidy Merselis[3], Joseph Naglich[4], John S. Sack[2], Ping Zhang[5], Chunshan Xie[4], Jeffrey Tredup[5], Laurel B. Stine[3], Cameron Messier[3], David L. Hope[3], Janet Caceres-Cortes[1], Luciano Mueller[1], Alaric J. Dyckman[6], John A. Newitt[5], Asmita Choudhury[7], Stephen C. Wilson[3,†]

Affiliations:

[1]Drug Discovery Analytical, Lead Discovery and Optimization, Discovery & Development Sciences, Bristol Myers Squibb, Lawrenceville, NJ, 08648, USA.

[2]Structural Biology, Lead Discovery and Optimization, Discovery & Development Sciences, Bristol Myers Squibb, Lawrenceville, NJ, 08648, USA.

[3]Discovery Immunology, Bristol Myers Squibb, 250 Water St. Cambridge, MA, 02141 USA.

[4]Mechanistic Pharmacology, Discovery & Development Sciences, Bristol Myers Squibb, Lawrenceville, NJ, 08648, USA.

[5]Protein Science, Lead Discovery and Optimization, Discovery & Development Sciences, Bristol Myers Squibb, Lawrenceville, NJ, 08648, USA.

[6]Immunology Chemistry, Discovery & Development Sciences, Bristol Myers Squibb, Lawrenceville, NJ, 08648, USA.

[7]Biocon-Bristol Myers Squibb Research and Development Center, Biocon Park, Plot No. 2 & 3, Bommasandra Phase IV, Jigani Link Road, Bangalore 560099, India

[†]Corresponding authors: tao.xie@bms.com and scwilson@gmail.com

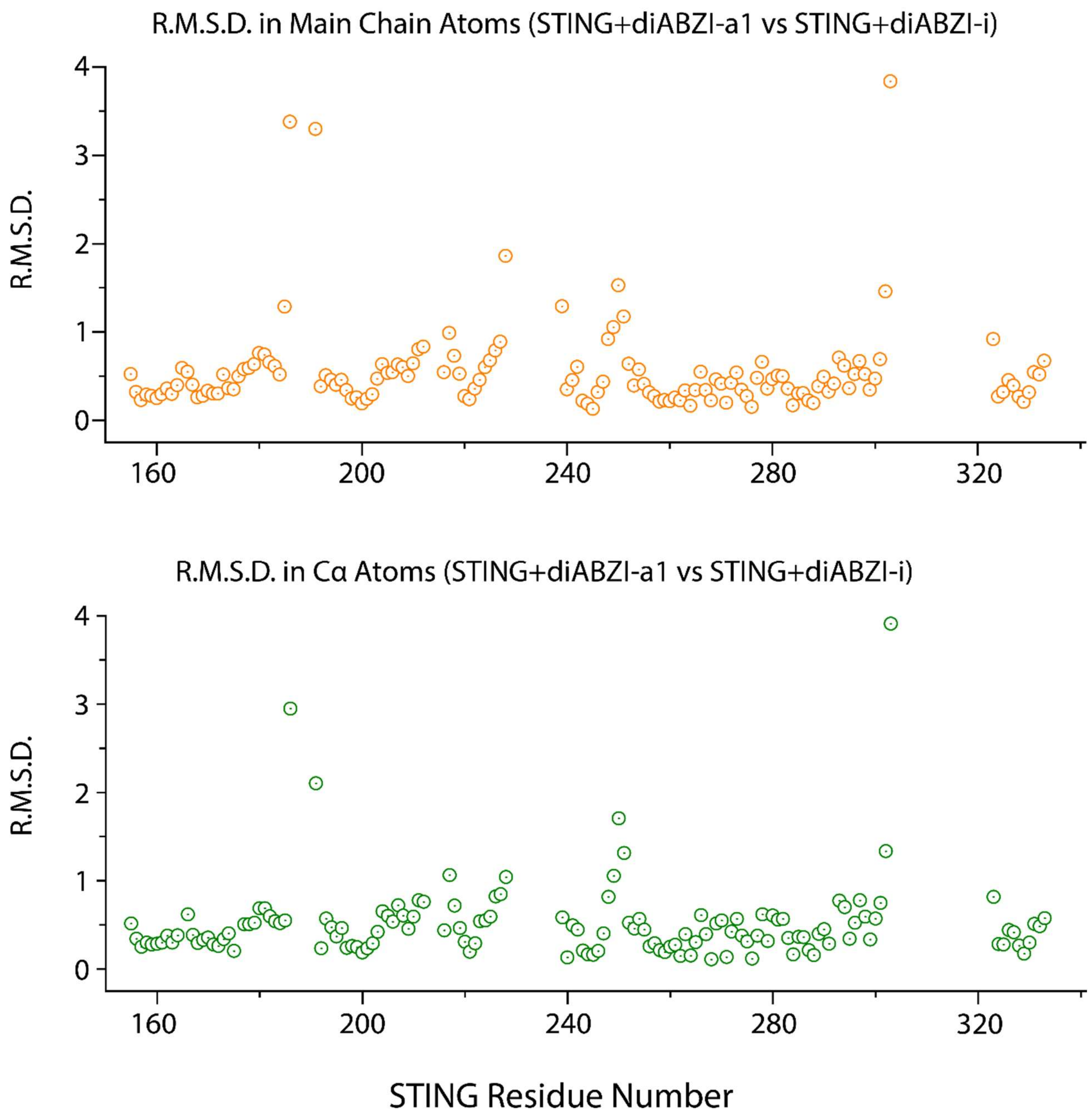

Supplementary Fig. 1. Root mean squared deviation (R.M.S.D.) between diABZI-i and diABZI-a1 of main chain atoms and Cα atoms.

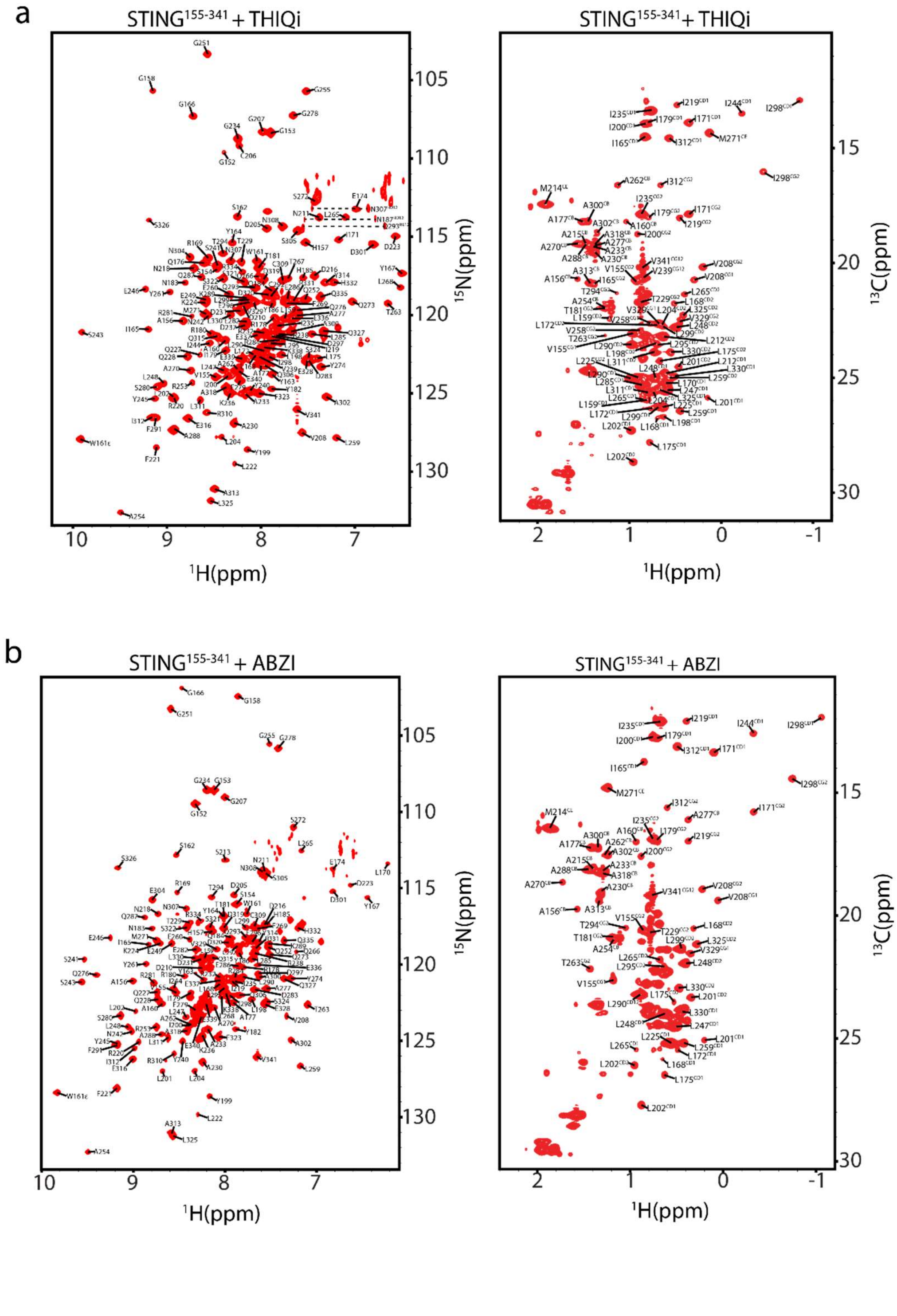

Supplementary Fig. 2. NMR spectra used for resonance assignments. **(a)** ^{1}H-^{15}N HSQC and ^{1}H-^{13}C HSQC spectra of STING$^{155-341}$-THIQi complex annotated with assignments. **(b)** ^{1}H-^{15}N HSQC and ^{1}H-^{13}C HSQC of ABZI-bound STING$^{155-341}$ annotated with assignments.

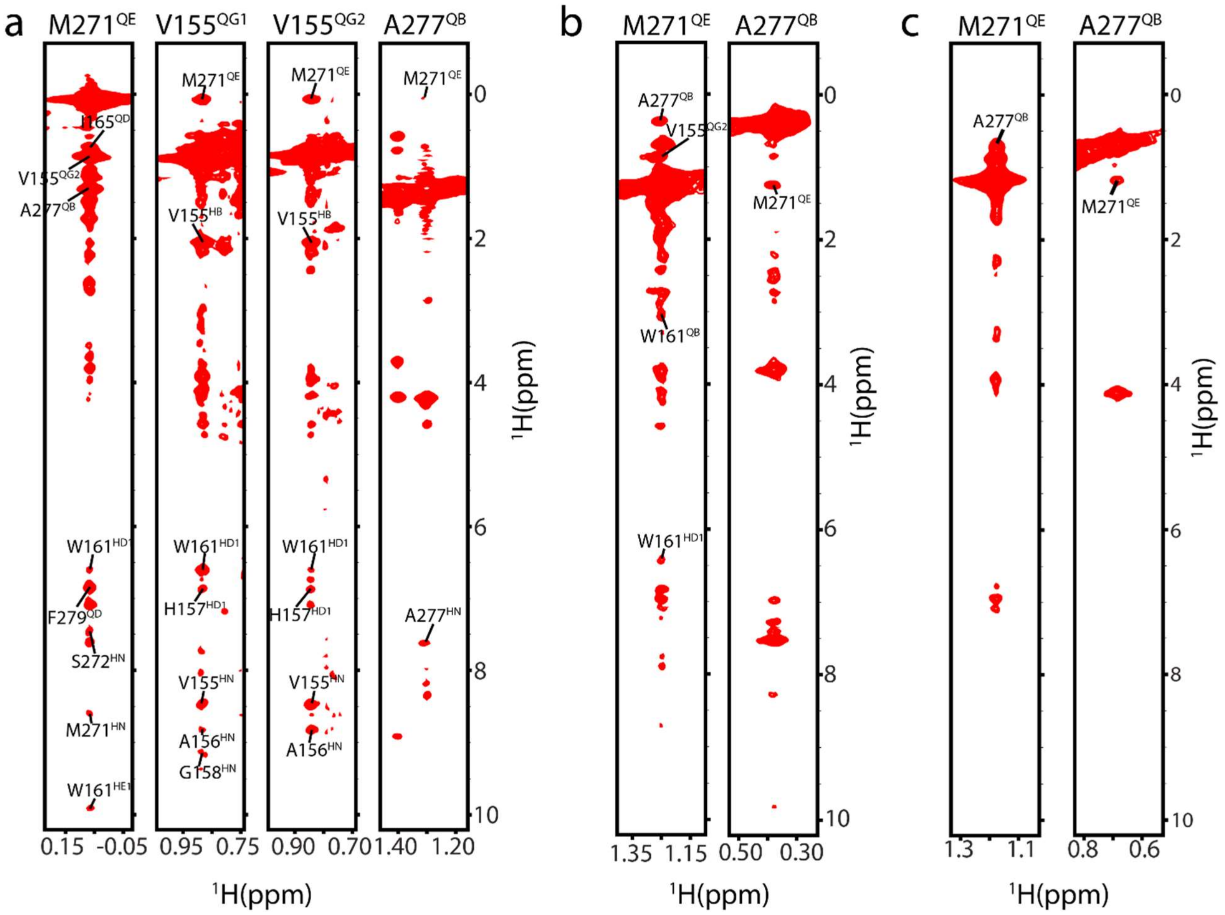

Supplementary Fig. 3. Selected strips of ^{13}C-edited NOESY-HSQC spectra of **(a)** THIQi-bound STING$^{155\text{-}341}$ highlighting the intramolecular NOEs between M271^{CH3} and other atoms, **(b)** ABZI-bound STING$^{155\text{-}341}$ showing NOEs between M271^{CH3} and other atoms, and **(c)** diABZI-a1-bound STING$^{155\text{-}341}$ confirming the assignment of A277^{CH3}.

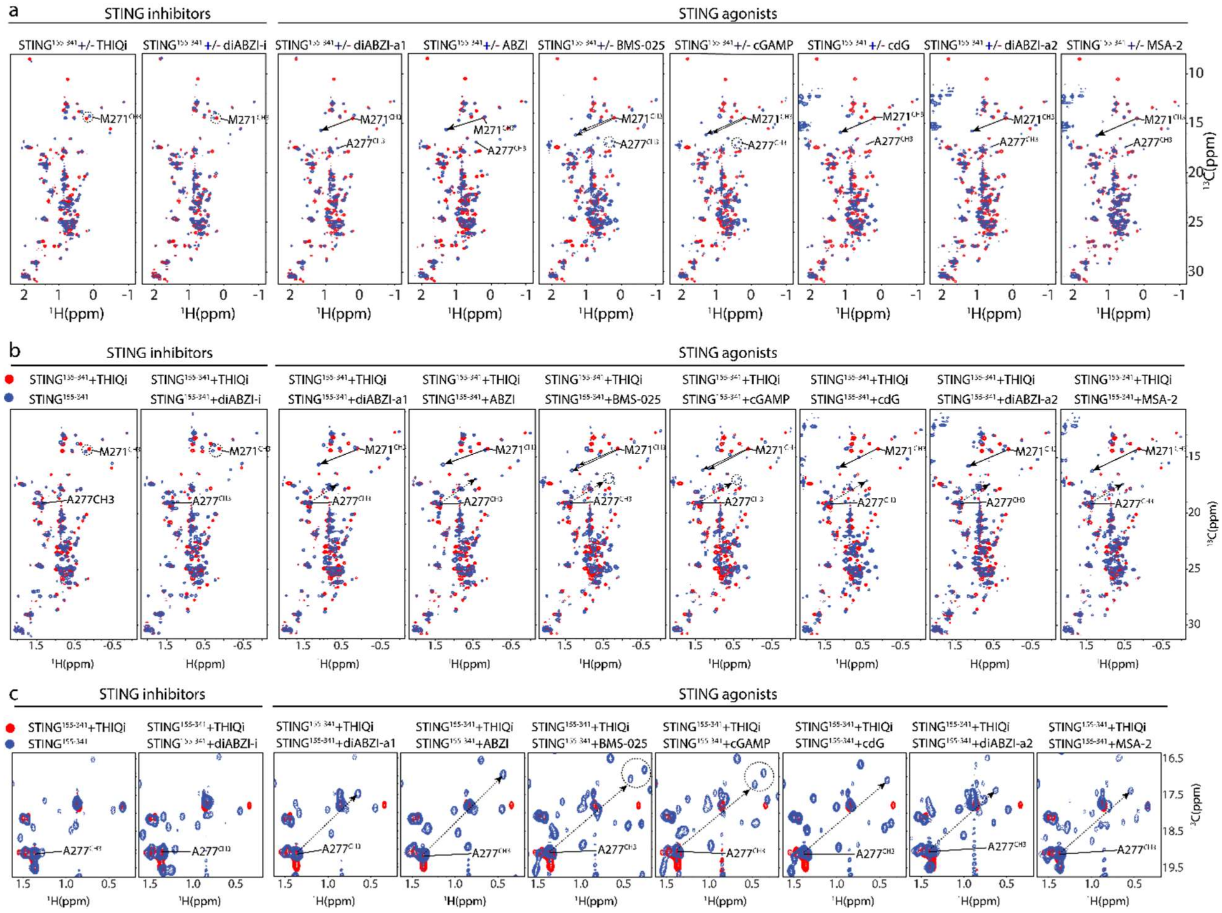

Supplementary Fig. 4. ^{1}H-^{13}C HSQC overlay of **(a)** STING$^{155\text{-}341}$ recorded in the absence and presence of various inhibitors and agonists, **(b)** STING$^{155\text{-}341}$-THIQi and STING$^{155\text{-}341}$ recorded in the presence of various inhibitors and agonists, and **(c)** Expanded view of STING$^{155\text{-}341}$-THIQi and STING$^{155\text{-}341}$ recorded in the presence of various inhibitors and agonists highlighting A277^{CH3}.

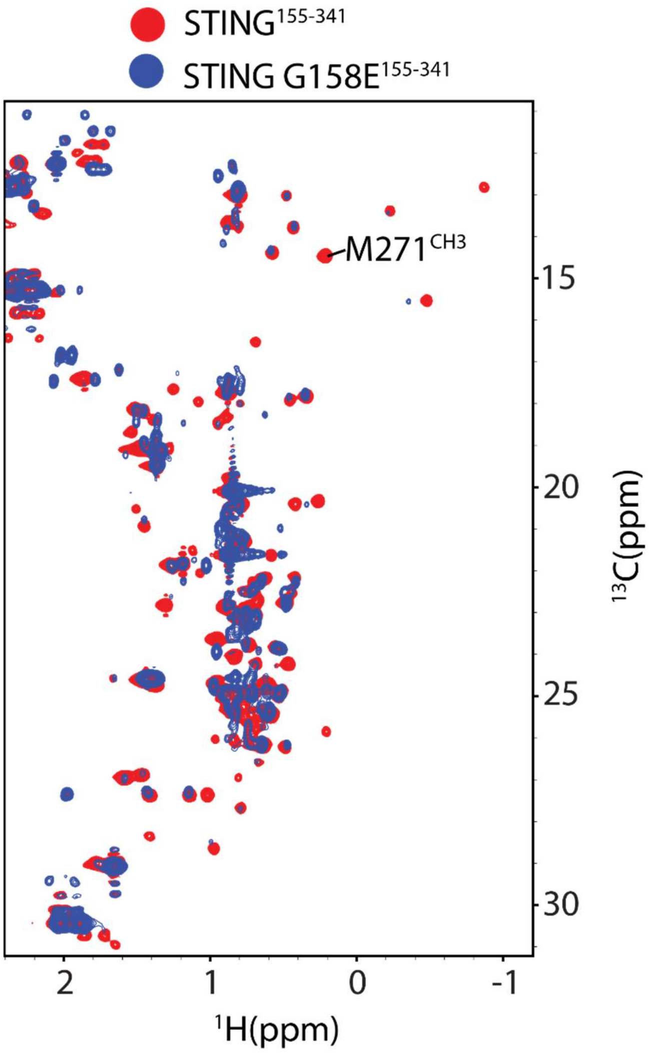

Supplementary Fig. 5. ^{1}H-^{13}C HSQC spectrum comparison between STING$^{155\text{-}341}$ (red) and STING$^{155\text{-}341}$ G158E (blue).

a

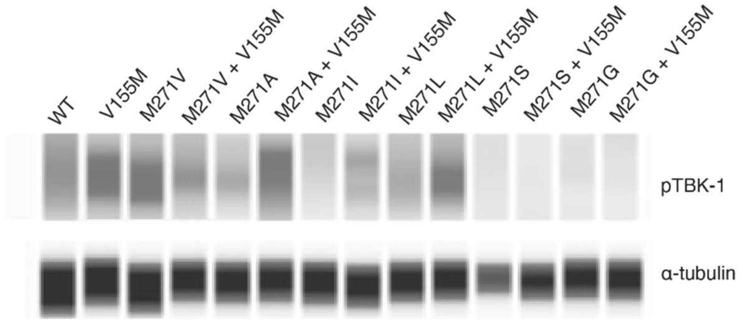

b

WT	V155M	M271V	M271V + V155M	M271A	M271A + V155M	M271I	M271I + V155M	M271L	M271L + V155M	M271S	M271S + V155M	M271G	M271G + V155M
1.0	1.7	1.5	1.6	1.1	2.2	NA	1.3	1.5	2.0	NA	NA	NA	NA

c

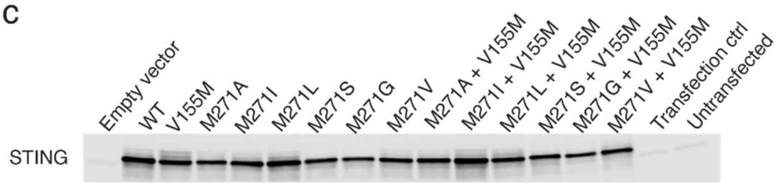

Supplementary Fig. 6. pTBK1 Jess western analysis **(a)** pTBK1 Jess western of all M271 mutants by transient transfection. **(b)** Relative quantitation of pTBK1 bands from **(a)**. **(c)** STING expression with transiently transfected constructs as detected by western blot.

Supplementary Table 1. X-Ray Data Collection, Refinement, and Superposition Statistics.

	**STING$^{155\text{-}341}$ + diABZI-a1**	**STING$^{155\text{-}341}$ + diABZI-i**
Crystal Parameters		
Space group	$P2_1$	$P2_12_12$
Cell dimensions	a = 35.97 Å, b = 72.64 Å, c = 66.43 Å, β = 98.02°	a = 80.07 Å, b = 122.95 Å, c = 36.45 Å
Molecules per AU[a]	2	2
Data Collection		
Beamline	NSLS-II 17ID-1 "AMX"	APS 17ID "IMCA-CAT"
Wavelength (Å)	0.92	1.0
Resolution range (Å)[b]	65.78–2.11 (2.15–2.11)	67.10–1.70 (1.73–1.70)
Observed / unique[c] reflections	62451 / 18081 (3349 / 953)	144067 / 34480 (8545 / 1997)
Completeness (%)[b]	92.2 (99.5)	84.8 (99.0)
R_{merge} (%)[b,d]	0.072 (0.522)	0.058 (0.724)
Refinement		
Resolution (Å)	25.43–2.11	21.53–1.70
R_{work} / R_{free}[e]	26.31 / 28.72	22.56 / 25.78
No. atoms		
Protein	5001	4821
Ligand	94	116
Water	106	309
B-factors		
Protein	50.90	20.88
Ligand	39.30	19.12
Water	45.95	37.52
r.m.s.d.[f]		
Bond lengths (Å)	0.007	0.008
Bond angles (°)	0.84	0.91
Ramachandran (%)[g]	96.18 / 2.55 / 1.27	97.90 / 1.05 / 1.05
PDB accession code	9CUB	9CUD
Superposition		
r.m.s.d. for main chain atoms (Å^2)	0.548	
r.m.s.d. for Cα atoms (Å^2)	0.511	

[a]Asymmetric unit. [b]Values in parentheses for resolution range, completeness, R_{merge}, and $I/\sigma(I)$ correspond to the last resolution shell. [c]Friedel pairs were treated as identical reflections. [d]$R_{merge}(I) = \sum_{hkl}\sum_j|[I(hkl)_j - I(hkl)]|/\sum_{hkl}I_{hkl}$, where $I(hkl)_j$ is the measurement of the intensity of reflection hkl and $\langle I(hkl)\rangle$ is the average intensity. [e]$R = \sum_{hkl}||F_{obs}| - |F_{calc}||/\sum_{hkl}|F_{obs}|$, where R_{free} is calculated without a σ cutoff for a randomly chosen 5% of reflections, which were not used for structure refinement, and R_{work} is calculated for the remaining reflections. [f]Root mean square deviations from ideal bond lengths/angles. [g]Number of residues in favored / additionally allowed / outlier regions.

	STING$^{155-341}$ + THIQi	STING$^{155-341}$ + cGAMP
Crystal Parameters		
Space group	$P2_1$	$P4_1$
Cell dimensions	a = 66.53 Å, b = 70.59 Å, c = 119.83 Å, β = 94.23°	a = b = 109.81 Å, c = 35.36 Å
Molecules per AU[a]	6	2
Data Collection		
Beamline	APS 17ID "IMCA-CAT"	APS 17ID "IMCA-CAT"
Wavelength (Å)	1.0	1.0
Resolution range (Å)[b]	60.778–2.71 (2.76–2.71)	49.11–2.22 (2.34–2.22)
Observed / unique[c] reflections	102768 / 30122 (4199 / 1418)	139081 / 21288 (21295 / 3077)
Completeness (%)[b]	99.1 (94.9)	99.7 (100.0)
R_{merge} (%)[b,d]	0.109 (0.525)	0.054 (0.719)
Refinement		
Resolution (Å)	17.92–2.71	23.66–2.22
R_{work} / R_{free}[e]	29.6 / 31.9	20.0 / 23.1
No. atoms		
Protein	7171	2584
Ligand	210	45
Water	73	43
B-factors		
Protein	24.92	62.15
Ligand	10.75	39.39
Water	16.73	59.82
r.m.s.d.[f]		
Bond lengths (Å)	0.008	0.010
Bond angles (°)	0.95	1.13
Ramachandran (%)[g]	96.09 / 2.96 / 0.95	96.95 / 1.83 / 1.22
PDB accession code	9CUC	9CUA

[a]Asymmetric unit. [b]Values in parentheses for resolution range, completeness, R_{merge}, and $I/\sigma(I)$ correspond to the last resolution shell. [c]Friedel pairs were treated as identical reflections. [d]$R_{\text{merge}}(I) = \sum_{hkl}\sum_j|[I(hkl)_j - I(hkl)]|/\sum_{hkl}I_{hkl}$, where $I(hkl)_j$ is the measurement of the intensity of reflection hkl and $\langle I(hkl)\rangle$ is the average intensity. [e]$R = \sum_{hkl}||F_{\text{obs}}| - |F_{\text{calc}}||/\sum_{hkl}|F_{\text{obs}}|$, where R_{free} is calculated without a σ cutoff for a randomly chosen 5% of reflections, which were not used for structure refinement, and R_{work} is calculated for the remaining reflections. [f]Root mean square deviations from ideal bond lengths/angles. [g]Number of residues in favored / additionally allowed / outlier regions.

	$STING^{155\text{-}341}$ + ABZI
Crystal Parameters	
Space group	$P2_12_12$
Cell dimensions	a = 93.10 Å, b = 130.17 Å, c = 35.92 Å
Molecules per AU[a]	2
Data Collection	
Beamline	APS 17ID "IMCA-CAT"
Wavelength (Å)	1.0
Resolution range (Å)[b]	75.73–1.96 (2.22–1.96)
Observed / unique[c] reflections	113224 / 21574 (2319 / 1079)
Completeness (%)[b]	91.7 (49.2)
R_{merge} (%)[b,d]	0.100 (0.558)
Refinement	
Resolution (Å)	16.40–1.96
R_{work} / R_{free}[e]	30.7 / 33.4
No. atoms	
Protein	2422
Ligand	39
Water	23
B-factors	
Protein	36.42
Ligand	14.79
Water	30.02
r.m.s.d.[f]	
Bond lengths (Å)	0.008
Bond angles (°)	0.96
Ramachandran (%)[g]	91.67 / 4.00 / 4.33
PDB accession code	9CUE

[a]Asymmetric unit. [b]Values in parentheses for resolution range, completeness, R_{merge}, and $I/\sigma(I)$ correspond to the last resolution shell. [c]Friedel pairs were treated as identical reflections. [d]$R_{merge}(I) = \sum_{hkl}\sum_j |[I(hkl)_j - I(hkl)]| / \sum_{hkl} I_{hkl}$, where $I(hkl)_j$ is the measurement of the intensity of reflection hkl and $\langle I(hkl)\rangle$ is the average intensity. [e]$R = \sum_{hkl}||F_{obs}| - |F_{calc}|| / \sum_{hkl}|F_{obs}|$, where R_{free} is calculated without a σ cutoff for a randomly chosen 5% of reflections, which were not used for structure refinement, and R_{work} is calculated for the remaining reflections. [f]Root mean square deviations from ideal bond lengths/angles. [g]Number of residues in favored / additionally allowed / outlier regions.

Supplementary Table 2. Structural properties of STING.

	PDB	Res. Num.	open/ closed	beta sheet lid	regulation status	Dimer interface
hSTING	4EF5	139-379	open	no	inactive	S_1
hSTING + c-di-GMP	4EF4	139-379	open	no	active	S_1
hSTING	4F9E	139-379	open	no	inactive	S_1
hSTING + c-di-GMP	4F9G	139-379	open	no	active	S_1
hSTING	4EMU	155-341	open	no	inactive	S_1
hSTING + c-di-GMP	4EMT	155-341	open	no	active	S_1
$hSTING^{H232R}$	4F5W	149-379	open	no	inactive	S_1
$hSTING^{H232R}$ + c-di-GMP	4F5Y	149-379	open	no	active	S_1
$hSTING^{G230A/H232R}$	4F5E	140-379	open	no	inactive	S_1
$hSTING^{G230A/H232R}$ + c-di-GMP	4F5D	140-379	closed	yes	active	S_2
$hSTING^{H232R}$ + compound 1(ABZI)	6DXG	149-379	open	no	active	S_2
$hSTING^{H232R}$ + compound 2(diABZI)	6DXL	149-379	open	no	active	S_2
$hSTING^{H232R}$ + 2',3'-cGAMP	4KSY	139-379	closed	yes	active	S_3
hSTING + 2',3'-cGAMP	4LOH	155-341	closed	yes	active	S_3
$hSTING^{G230A/H232R}$ + 2',3'-cGAMP	6DNK	133-379	closed	yes	active	S_2
$hSTING^{G230A/H232R}$ + c-di-AMP	6CY7	133-379	closed	yes	active	S_3
$hSTING^{H232R}$ + c-di-AMP	6CFF	133-379	closed	yes	active	S_2
hSTING + SHR1032	7T9V	153-343	open	no	active	S_1
hSTING + SHR169224	7T9U	153-343	open	no	active	S_1
$hSTING^{H232R}$ + compound 11	7SSM	154-335	closed	yes	active	S_2
$hSTING^{G230A/H232R/R293Q}$ + MK-1454	7MHC	154-336	closed	yes	active	S_3
$hSTING^{H232R}$ + agonist 15	8STH	153-339	closed	no	active	S_2
$hSTING^{H232R}$ +XMT-1616	8STI	153-338	closed	no	active	S_2
$hSTING^{G230A/H232R/R293Q}$ + compound 1	6MX3	154-336	open	no	inactive	S_1
$hSTING^{G230A/H232R/R293Q}$+ compound 18	6MXE	154-335	open	no	inactive	S_1
$hSTING^{H232R}$	6NT5	4-336	open	no	inactive	S_1
chSTING+2',3'-cGAMP	6NT7	11-342	closed	yes	active	S_3
hSTING+ HB3089	8GT6	5-337	open	no	active	S_1
$hSTING^{G230A/R293Q}$ +diABZI-i	9CUD	155-341	open	no	inactive	S_3
$hSTING^{G230A/R293Q}$ +diABZI-a1	9CUB	155-341	open	no	active	S_3
$hSTING^{G230A/R293Q}$ +THIQi	9CUC	155-341	open	no	inactive	S_1
$hSTING^{G230A/R293Q}$+2',3'-cGAMP	9CUA	155-341	closed	yes	active	S_2
$hSTING^{H232R}$+ABZI	9CUE	155-341	open	no	active	S_3

Uncropped Westerns

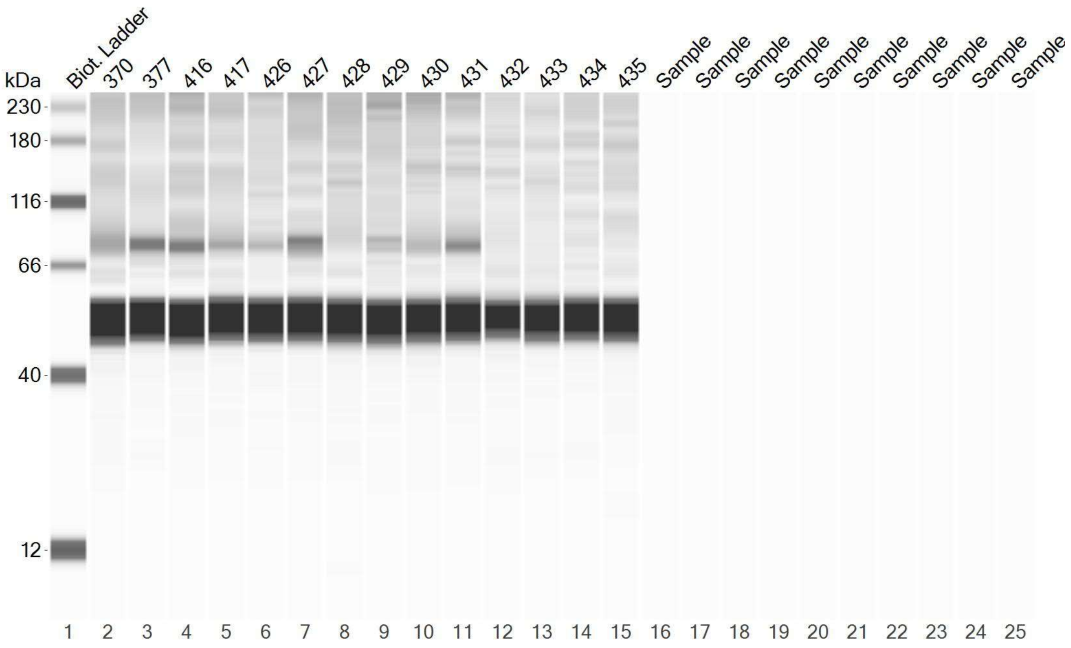

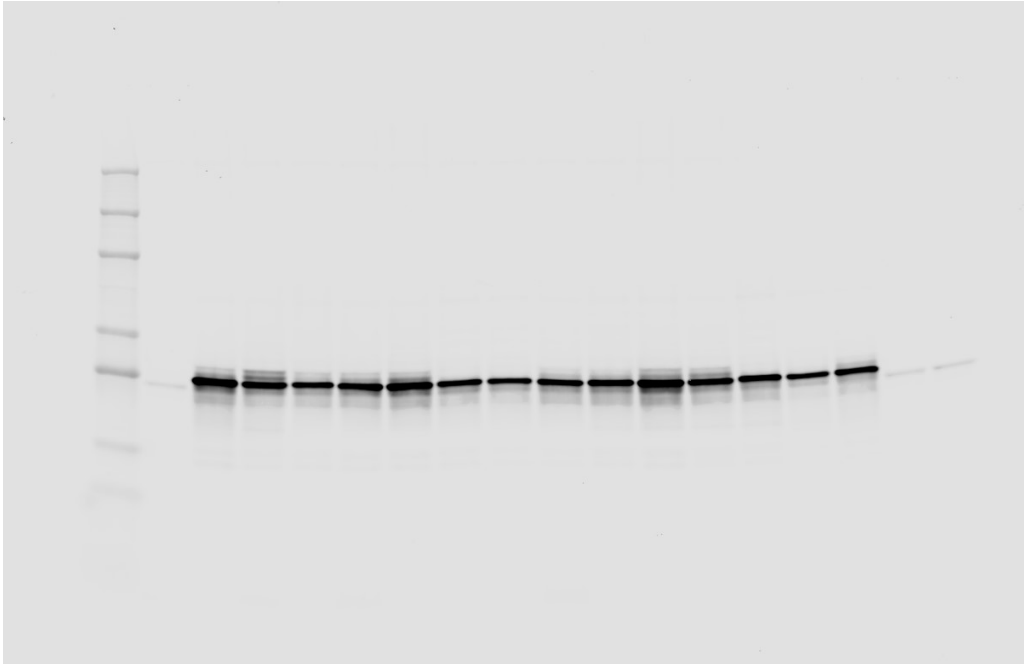

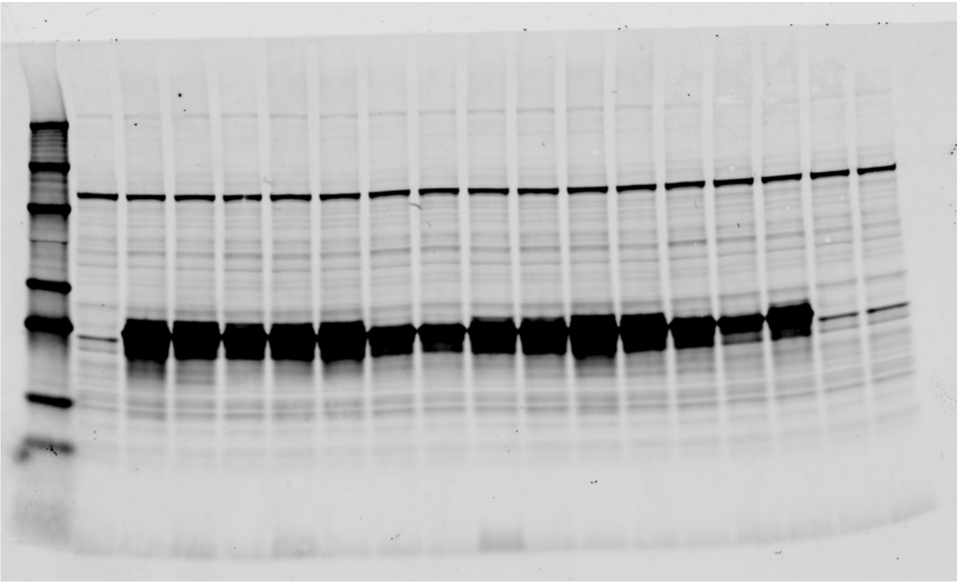

Supplement: Supplementary file 1 — Supplementary Information [file 41467_2025_60632_MOESM1_ESM.pdf]
