# Supplementary material for: Orthosteric STING inhibition elucidates molecular correction of SAVI STING
Source: Nat Commun. 2025 Jul 1;16:5695. doi: 10.1038/s41467-025-60632-5 (PMC12217682; doi:10.1038/s41467-025-60632-5)
Supplement: Supplementary file 2 — Reporting Summary [file 41467_2025_60632_MOESM2_ESM.pdf]

nature portfolio

Corresponding author(s): Stephen Wilson

Last updated by author(s): 5/2/2025

Reporting Summary

Nature Portfolio wishes to improve the reproducibility of the work that we publish. This form provides structure for consistency and transparency in reporting. For further information on Nature Portfolio policies, see our Editorial Policies and the Editorial Policy Checklist.

Statistics

For all statistical analyses, confirm that the following items are present in the figure legend, table legend, main text, or Methods section.

n/a	Confirmed	
☐	☒	The exact sample size (*n*) for each experimental group/condition, given as a discrete number and unit of measurement
☐	☒	A statement on whether measurements were taken from distinct samples or whether the same sample was measured repeatedly
☐	☒	The statistical test(s) used AND whether they are one- or two-sided<br>*Only common tests should be described solely by name; describe more complex techniques in the Methods section.*
☒	☐	A description of all covariates tested
☐	☒	A description of any assumptions or corrections, such as tests of normality and adjustment for multiple comparisons
☐	☒	A full description of the statistical parameters including central tendency (e.g. means) or other basic estimates (e.g. regression coefficient) AND variation (e.g. standard deviation) or associated estimates of uncertainty (e.g. confidence intervals)
☐	☒	For null hypothesis testing, the test statistic (e.g. *F*, *t*, *r*) with confidence intervals, effect sizes, degrees of freedom and *P* value noted<br>*Give P values as exact values whenever suitable.*
☒	☐	For Bayesian analysis, information on the choice of priors and Markov chain Monte Carlo settings
☒	☐	For hierarchical and complex designs, identification of the appropriate level for tests and full reporting of outcomes
☒	☐	Estimates of effect sizes (e.g. Cohen's *d*, Pearson's *r*), indicating how they were calculated

Our web collection on statistics for biologists contains articles on many of the points above.

Software and code

Policy information about availability of computer code

Data collection	Not applicable
Data analysis	Not applicable

For manuscripts utilizing custom algorithms or software that are central to the research but not yet described in published literature, software must be made available to editors and reviewers. We strongly encourage code deposition in a community repository (e.g. GitHub). See the Nature Portfolio guidelines for submitting code & software for further information.

Data

Policy information about availability of data

All manuscripts must include a data availability statement. This statement should provide the following information, where applicable:

- Accession codes, unique identifiers, or web links for publicly available datasets
- A description of any restrictions on data availability
- For clinical datasets or third party data, please ensure that the statement adheres to our policy

Source data is available with this paper as a Source Data file. Crystallographic structure data (atomic coordinates and structure factors) have been deposited into the Protein Data Bank (https://www.rcsb.org) and are publicly available as of the date of publication. Accession numbers are: 9CUA (Human STING G230A/R293Q variant bound to cGAMP); 9CUB (Human STING G230A/R293Q variant bound to diABZI-a1); 9CUC (Human STING G230A/R293Q variant bound to THIQi); 9CUD (Human STING G230A/R293Q variant bound to diABZI-i); and 9CUE (Human STING H232R variant bound to ABZI). NMR chemical shift assignments of THIQi-complexed and ABZI-complexed STING G230A/R293Q variant have been deposited in the BMRB database (https://bmrb.io) under accession code 52522 and 52528

respectively. The data are available as of the date of publication. The PDB codes of the previously published structures used in this study are 4LOH, 4EMU, 4EMT, 4F5Y, 4EF5, 4EF4, 4F9E, 4F9G, 4F5W, 4F5E, 4F5D, 6DXG, 6DXL, 4KSY, 6DNK, 6CY7, 6CFF, 7T9V, 7T9U, 7SSM, 7MHC, 8STH, 8STI, 6MX3, 6MXE, 6NT5, 6NT7, 8GT6.

Research involving human participants, their data, or biological material

Policy information about studies with human participants or human data. See also policy information about sex, gender (identity/presentation), and sexual orientation and race, ethnicity and racism.

Reporting on sex and gender	Information on donor sex and gender is included in the Methods section. Peripheral blood samples collected from healthy volunteers were obtained following institutional guidelines, and written informed consent was provided by each volunteer prior to participation. Age, sex and/or gender was not considered in the study design because this could have led to prohibitive donor identification and medical privacy breach. Participants were compensated $50- or $100-dollars USD, depending on the size of the donation. All procedures involving human samples complied with ethical standards and guidelines approved by the Western Copernicus Group Institutional Review Board (WCGIRB) under "Standard Operating Procedure for Obtaining Venous Blood and Other Non-Invasive Biological Specimens for Research Purposes for Bristol-Myers Squibb Research" (WIRB protocol #20161208).
Reporting on race, ethnicity, or other socially relevant groupings	Race, ethnicity or other social relevant groupings were not considered during the collection of peripheral blood samples.
Population characteristics	No population characteristics were controlled in the selection of healthy volunteers for this study (see above).
Recruitment	Donors volunteered to solicitations sent out on by email with the knowledge that their blood would be collected for scientific purposes. Participants were compensated $50- or $100-dollars USD, depending on the size of the donation.
Ethics oversight	All research contained with this article complies with rules and regulations maintained by Bristol Myers Squibb. All procedures involving human samples complied with ethical standards and guidelines approved by the Western Copernicus Group Institutional Review Board (WCGIRB) under "Standard Operating Procedure for Obtaining Venous Blood and Other Non-Invasive Biological Specimens for Research Purposes for Bristol-Myers Squibb Research" (WIRB protocol #20161208).

Note that full information on the approval of the study protocol must also be provided in the manuscript.

Field-specific reporting

Please select the one below that is the best fit for your research. If you are not sure, read the appropriate sections before making your selection.

[x] Life sciences [ ] Behavioural & social sciences [ ] Ecological, evolutionary & environmental sciences

For a reference copy of the document with all sections, see nature.com/documents/nr-reporting-summary-flat.pdf

Life sciences study design

All studies must disclose on these points even when the disclosure is negative.

Sample size	No statistical method was used to predetermine sample sizes. However, biological replicates of 3 or higher were typically employed in this study.
Data exclusions	No data exclusions to report.
Replication	Reproducibility was evaluated by performing at least 3 biological replicates unless otherwise stated in the Figure legends. All attempts at replication were successful.
Randomization	Throughout data acquisition, all experiments samples were randomized. However for studies using uniform biological material (e.g. cell lines), randomization was not relevant.
Blinding	Blinding was not possible for in vitro experiments because these experiments required investigators to be aware of the experimental conditions before execution.

Reporting for specific materials, systems and methods

We require information from authors about some types of materials, experimental systems and methods used in many studies. Here, indicate whether each material, system or method listed is relevant to your study. If you are not sure if a list item applies to your research, read the appropriate section before selecting a response.

Materials & experimental systems

n/a	Involved in the study	
	x	Antibodies
	x	Eukaryotic cell lines
x		Palaeontology and archaeology
x		Animals and other organisms
x		Clinical data
x		Dual use research of concern
x		Plants

Methods

n/a	Involved in the study	
x		ChIP-seq
x		Flow cytometry
x		MRI-based neuroimaging

Antibodies

Antibodies used	The following commercial antibodies were used. Catalog, clone and lot numbers are provided wherever such information is publicly available. Antibody dilutions are provided in the Methods section.<br>1) STING (CST, #13647, D2P2F)<br>2) TBK-1 (CST, #5483, D52C2)<br>3) Vinculin (Santa Cruz, sc-73614, 7F9)<br>4) alpha-tubulin (Novus Biologicals, NB100-690, DM1A)
Validation	Validation statements for all antibodies listed above can be found through the following links to the manufacturer's website:<br>1) https://www.cellsignal.com/products/primary-antibodies/sting-d2p2f-rabbit-mab/13647?srsltid=AfmBOortHJ0RdAZz0v6THyV1cG9T0zlL6ja64D-CojzRkCkfqQCSWP-t<br>2) https://www.cellsignal.com/products/primary-antibodies/phospho-tbk1-nak-ser172-d52c2-xp-rabbit-mab/5483?srsltid=AfmBOor6PNnKFeJT8lP22txyxHNdUcWHa4Oui7qpGaFNzc-0euG2NQC2<br>3) https://www.scbt.com/p/vinculin-antibody-7f9?srsltid=AfmBOopGfZVuklpu3aXGOupSKedPr322JI9BwpgQgSWD07bGFdOFESCb<br>4) https://www.novusbio.com/products/alpha-tubulin-antibody-dm1a_nb100-690

Eukaryotic cell lines

Policy information about cell lines and Sex and Gender in Research

Cell line source(s)	Cell lines (HEK293, THP-1s) were purchased from Invivogen. The respective catalog numbers are provided in the Methods section.
Authentication	None of the cell lines were authenticated internally
Mycoplasma contamination	All cell lines tested negative for Mycoplasma on a quarterly testing schedule
Commonly misidentified lines (See ICLAC register)	No commonly misidentified cell lines were used in the study

Plants

Seed stocks	Not applicable
Novel plant genotypes	Not applicable
Authentication	Not applicable
